# Announcing the *International Journal of Molecular Sciences* Young Investigator and Travel Awards 2017

**DOI:** 10.3390/ijms18040791

**Published:** 2017-04-08

**Authors:** International Journal of Molecular Sciences Editorial Office

**Affiliations:** MDPI AG, St. Alban-Anlage 66, 4052 Basel, Switzerland; ijms@mdpi.com

With the goal of recognizing outstanding contributions to the field of molecular sciences by young investigators under the age of 40 (by 31 December 2016), and early-career investigators, which includes postdoctoral students and PhD students, and assisting the early-career investigators in attending international conferences in 2017, last year the *International Journal of Molecular Sciences* accepted nominations for Young Investigator and Travel Awards 2017. Over 200 nominations were received and were evaluated by a panel of judges comprised of *International Journal of Molecular Sciences* editorial board members.

We are excited to announce the winner of the Young Investigator award: Dr. Rob W.J. Collin who will be awarded 2000 Swiss Francs; and the following winners for Travel Awards, Dr. Yi Ma, Dr. Miranda Ween, and Dr. Reza M. Zadegan who will be supported with up to 800 Swiss Francs each towards their travel expenses to attend international conferences in 2017.

## 1. Young Investigator Awardee

**Figure:**
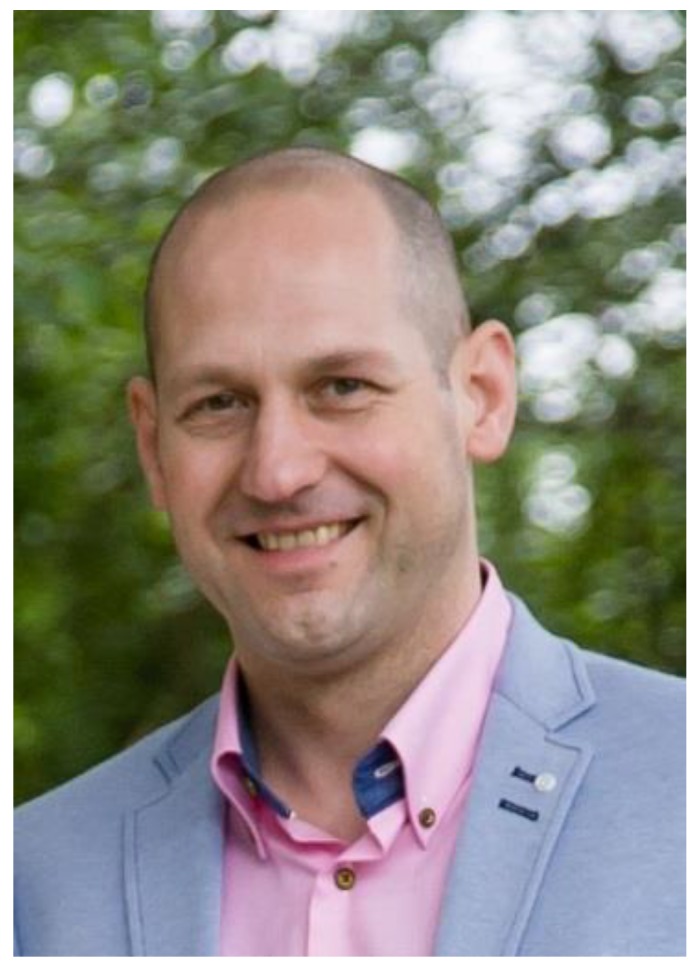
Dr. Rob W.J. Collin

Dr. Rob W.J. Collin is an Associate Professor at the Department of Human Genetics, Radboud University Medical Center in Nijmegen, The Netherlands, and is affiliated to the Donders Institute for Brain, Cognition and Behaviour. In 2000, Dr. Collin obtained his M.Sc. degree in Chemistry and in 2006, he successfully defended his doctoral thesis entitled “Exploring the role of Alzheimer’s amyloid-β precursor protein APP and its relatives in *Xenopus* intermediate pituitary, under the supervision of Prof. Dr. Gerard J.M. Martens. After obtaining his PhD degree, he changed gears to the field of Human Genetics, and as a post-doctoral researcher, he was involved in the identification of several novel disease-causing genes for inherited hearing impairment (*ESRRB*, *LRTOMT*) and inherited (vitreo)retinal diseases (*EYS*, *C2orf71*, *IMPG2*, *MVK*, *TSPAN12* and *ZNF408*). Since 2009, Dr. Collin has moved his scientific activities towards the development of novel genetic therapies for retinal diseases, in particular those focusing on correcting pre-mRNA splicing defects. Supported by a prestigious VENI grant from the Netherlands Organization for Scientific Research, and an Individual Investigator Award from the Foundation Fighting Blindness USA, amongst others, he and his group have developed an antisense oligonucleotide-based therapeutic strategy for the future treatment of *CEP290*-associated LCA. In 2010, he was a visiting scientist in the lab of Prof. Dr. Jean Bennett, at the F.M Kirby Center for Molecular Ophthalmology, Scheie Eye Institute in Philadelphia, PA, USA.

## 2. Travel Awards Awardees

### 2.1. Research Field: Biochemistry

**Figure:**
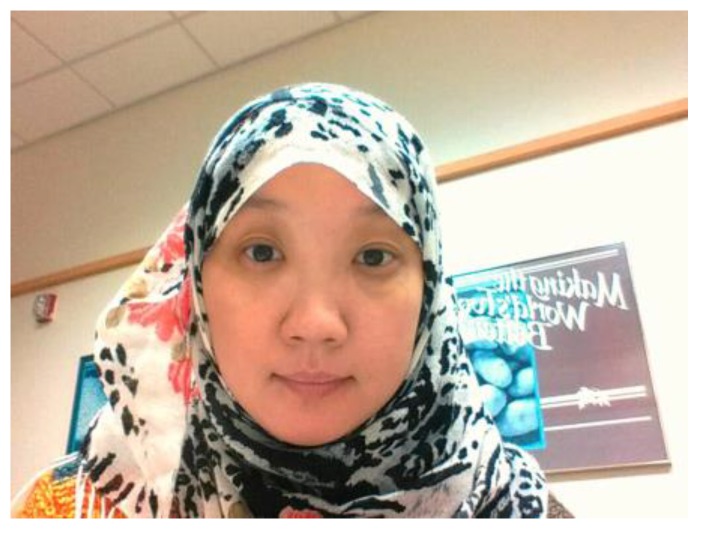
Dr. Yi Ma

Dr. Yi Ma, Post-Doctoral Research Scientist at University of Connecticut, USA. Dr. Ma obtained her Ph.D in Plant Biology from the Pennsylvania State University in 2008 under the supervision of Professor David Braun. Her Ph.D research was focused on carbohydrate partitioning in maize. She was working on the characterization of a tie-dyed1 (*tdy1*) mutant, which accumulates excess levels of sugars and starch in leaves. She cloned *Tie*-*dyed1*, a novel gene that expresses in phloem cells including sieve elements and companion cells. TDY1 is localized on the endoplasmic reticulum (ER) membranes involved in phloem unloading in maize. The findings provide insights into managing carbohydrate for yield improvement and biofuel production. Dr. Ma then switched to studying Ca^2+^ associated signaling in plant cells. She is working on identifying Ca^2+^ conducing channels that are involved in different signaling transduction pathways, such as BR signaling, plant innate immunity and meristem development. She has demonstrated that different Ca^2+^ sources could be responsible for different pathways. Dr. Ma is investigating Arabidopsis plants expressing an optogenetic cAMP sensor to monitor cAMP generation in responses to various environmental cues. 

### 2.2. Research Field: Molecular Medicine

**Figure:**
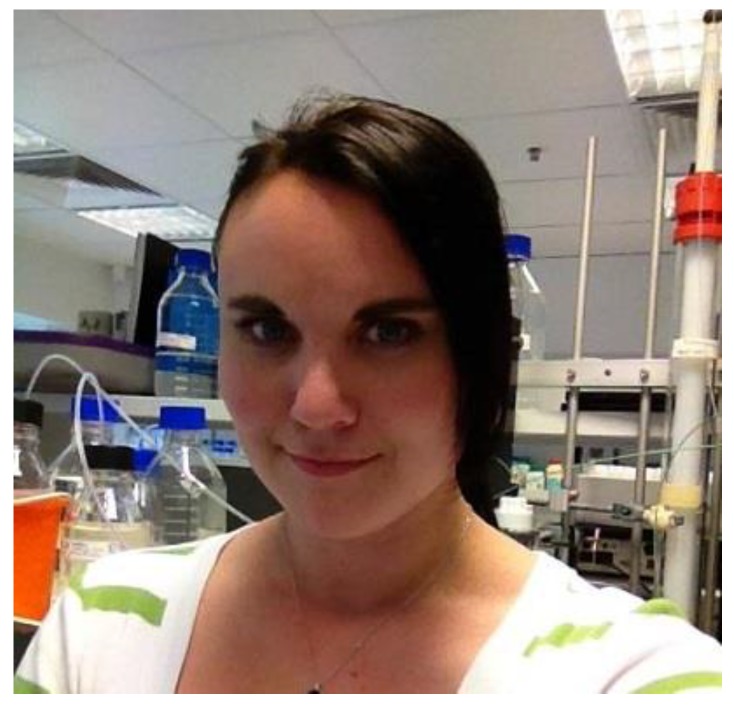
Dr. Miranda Ween

Dr. Ween completed her undergraduate Biomedical Science degree at the University of Adelaide, majoring in Human Physiology and Immunity and Infection. She was awarded her PhD in 2011 from the University of Adelaide, for a project performing highly complex and technically demanding cell biology studies into the role of protein interactions in ovarian cancer progression and received an award for her outstanding thesis. Dr Ween then joined the Research Centre for Infectious Diseases for her first postdoctoral role, investigating how lung bacteria survive in different conditions. Dr. Ween then joined the Lung Research Laboratory located in the Hanson Institute at the Royal Adelaide Hospital to study smoking related lung damage and susceptibility to infection. From there, Dr. Ween also expanded her research into investigating the effects E-cigarettes have on the lungs, with a particular focus on how their usage may affect the users’ immune cells’ ability to recognize pathogenic bacteria and phagocytose them. 

### 2.3. Research Field: Biomaterial

**Figure:**
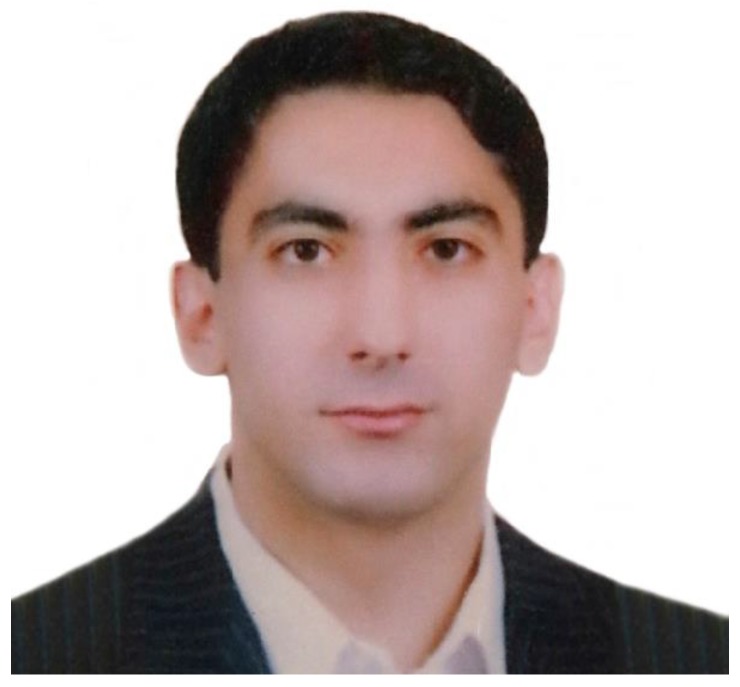
Dr. Reza M. Zadegan

To become a leading biomedical DNA nanotechnologist, Dr. Zadegan earned a B.Sc. in Biology-Cytology and Microbiology with a minor in Cell and Molecular Biology, an M.Sc. in Cell and Molecular Biology with a minor in bioelectronics, as well as a Ph.D. in Nanoscience with a minor in Molecular Biology. He completed graduate work under RNA/DNA bio-nanoscience pioneer, Prof. Jørgen Kjems, which led to the design, modeling, construction, and characterization of DNA nanostructures for biological and chemical applications. To gain interdisciplinary research skills, Dr. Zadegan then joined the Boise State University as a postdoctoral researcher in the Micron School of Materials Science and Engineering under the leadership of Dr. William L. Hughes whose team creates chemical reaction networks, nucleic acid memory, and lithographic masks using DNA as the engineering material. As a researcher in the laboratory of Dr. Hughes, he has been providing operational leadership in the Nucleic Acid Memory (NAM) group. Dr. Zadegan believes that nucleic acid-based materials are ideal candidates for interdisciplinary bio-nanomaterials science, and it is hence their coding, synthesis, and exploration of their unique properties that forms the basis of the research program he plans to establish. His current research focuses on synthesizing innovative biomaterials for a range of applications, with particular emphasis on nanostructured materials and devices and on developing alternative digital memory storage technologies given the likelihood that demand will outpace capacity and production of current technologies.

On behalf of the *International Journal of Molecular Sciences’* editorial board members and editorial staffs, we wish to congratulate these four outstanding scholars for their accomplishments.

## Prize Awarding Committee

The Editorial Board of the International Journal of Molecular Sciences.

